# Lead (Pb) in the tissues of *Anatidae*, *Ardeidae*, *Sternidae* and *Laridae* of the Northern Hemisphere: a review of environmental studies

**DOI:** 10.1007/s11356-019-04799-7

**Published:** 2019-03-21

**Authors:** Jan Korbecki, Izabela Gutowska, Dariusz Chlubek, Irena Baranowska-Bosiacka

**Affiliations:** 1 Department of Biochemistry and Medical Chemistry, Pomeranian Medical University, Powstańców Wlkp. 72 Av., 70-111 Szczecin, Poland; 2 Department of Biochemistry and Human Nutrition, Pomeranian Medical University, Broniewskiego 24 Str., 71-460 Szczecin, Poland

**Keywords:** Water bird, Heavy metal, Lead pollution, Gull, Egret, *Anatidae*, *Ardeidae*, *Laridae*

## Abstract

Due to the ability of birds to travel long distances in the air, the potential feeding area of each individual is much larger than that of typical terrestrial animals. This makes birds a convenient indicator of environmental lead (Pb) pollution over large areas, in particular areas of inland and coastal waters. The aim of this study was to assess the concentrations of Pb in various organs of water birds from a variety of locations. The focus was on ducks, geese and swans (*Anatidae*); herons and egrets (*Ardeidae*); terns (*Sternidae*); and gulls (*Laridae*). This article describes the level of lead in the most commonly studied tissue types: feathers, bones and the liver. The study also presents data concerning the concentration of lead in the eggs of water birds. The highest levels of lead pollution can be observed in China and Korea, related to their high level of industrialization. In Iran too, environmental lead pollution is high, likely due to the developed petrochemical industry. Lead pollution in Japan, as well as in Western European countries (Spain, France, Italy), seems to be much lower than in China, India or Iran. Nevertheless, the level of pollution in Europe is higher than satisfactory, despite the introduction of a number of bans related to, for example, the use of leaded petrol or lead-containing paints. Finally, the USA and Canada appear to be the areas with the lowest lead pollution, possibly due to their low population densities.

## Introduction

The ubiquity and toxicity of lead (Pb) have it ranked as the second most dangerous environmental poison in the world (ATSDR 2007 Substance Priority List). Its presence in the environment is closely tied to human activity. In the twentieth century, the amount of Pb in the environment from anthropogenic sources increased rapidly following the discovery of the anti-knock properties of lead tetraethyl in diesel engines and the use of lead in acid-lead batteries. Since the mid-twentieth century, leaded petrol was gradually withdrawn from use in most countries (between 1976 and 1986 in the USA, in the European Union the ban was introduced in 2005). Unfortunately, lead compounds are still used in aviation fuels, which, with increasing air traffic, constitute a serious problem. The sale of lead-based paints was banned in the USA in the late 1970s and in the EU in 1992, but lead-based paints can still be used in the renovation and maintenance of historic buildings, bridges and structures and works of art (EP 2005, 2009; EU 2008; Carr et al. 2011).

Currently, Pb is still used in the production of batteries, fishing sinkers and bullets for firearms (Haig et al. 2014; Goddard et al. 2008; Rattner et al. 2008). Pb ammunition and fishing tackle currently represent a substantial environmental problem due to their widespread use in recreational and subsistence activities in wildlife habitats. This problem is particularly relevant for birds because their mobility and diverse foraging strategies contribute to potential exposure and subsequent toxicological impairment in a broad array of species (Haig et al. 2014; United Nations Environment Programme 2011a, 2011b). Pb in bottom sediments of water reservoirs is also a threat to waders collecting food there, especially in industrialized and urbanized regions.

The aim of this study was to make a comparative analysis of Pb content in the liver, bones, eggs and feathers of birds living in the Northern Hemisphere on the basis of published data from the last 20 years (1998–2018). The available literature provided data on members of the families *Anatidae*, *Ardeidae*, *Sternidae* and *Laridae*. These are mainly small- and medium-sized species such as *Sternidae*, *Laridae* and ducks from the *Anatidae* family, which are more numerous and more diverse in behaviour and ecology than large birds (e.g. herons from the *Ardeidae* family or swans from the *Anatidae* family). Nevertheless, all these birds play essential roles in the functioning of the ecosystem and, due to their position in the trophic chain, they are susceptible to bioaccumulation of pollutants, including heavy metals. All individuals differ in body weight, metabolic rate, habitat, range and diet, and therefore, it can be expected that birds living in the same area will accumulate Pb in different amounts depending on these factors. Many of these birds can also live directly in or on the outskirts of cities (e.g. ducks, gulls). Moreover, many of them are hunted species, which makes them available for ecotoxicological studies (e.g. ducks).

With the wealth of available information using wild bird species as biomonitors, it should be possible to assess the state of the entire aquatic ecosystem of the Northern Hemisphere in terms of Pb pollution over of the last 20 years.

## Methods

**Fig. 1:**
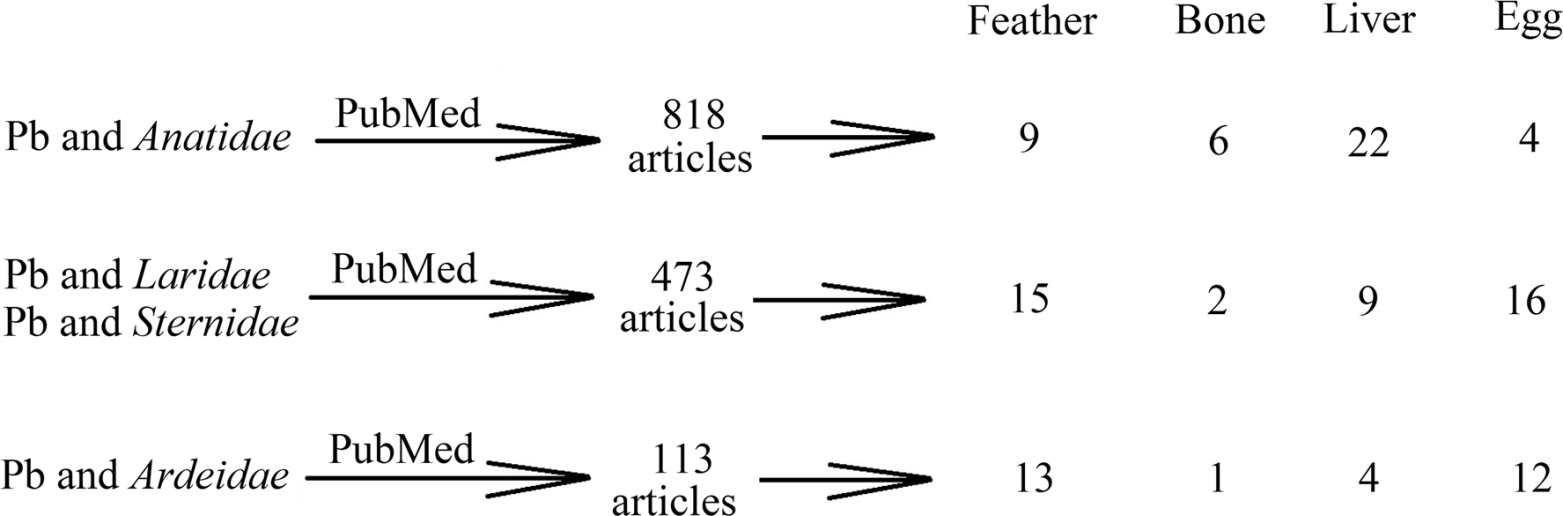
Selection of articles used to prepare this review paper. First of all, we searched PubMed for articles containing the word “lead” and the name of a given family of waterbirds in abstract, title or keywords. The paper was based on selected data, i.e. excluding incomplete data, those concerning other areas of research or found in previous papers. The fewest articles concerned the concentration of lead in waterbird bones, and the largest number was on the concentration of lead in feathers and the livers

The PubMed search engine (https://www.ncbi.nlm.nih.gov/pubmed/) was used to search for all articles on a given topic (Fig.1). The search was carried out in order to search for words appearing in the title, abstract or keywords. The following phrases were introduced, to find articles containing the word “lead” and representatives of groups of searched bird families:

- *Anatidae*: “(Pb or lead) and (duck or goose or mallard or anatidae or swan or cygnus or eider or anas)”
- *Sternidae* and *Laridae*: “(Pb or lead) and (gull or larus or tern or sterna)”
- *Ardeidae*: “(Pb or lead) and (heron or egret)”

We selected articles researching the state of the natural environment on the basis of the article title. In our review, we focused mainly, but not exclusively, on data from the last 20 years (1998–2018) to present the levels of lead in bones, feathers, the liver and eggs of families of waterbirds living in the Northern Hemisphere.

## Results

### Lead in the feathers of water birds

Feathers provide thermal insulation and protect against mechanical injuries. Furthermore, they ensure an appropriate aerodynamic surface for flying. Feathers are made of proteins rich in sulphur-containing amino acids, which is why they accumulate heavy metals (Burger and Gochfeld 1992; Lodenius and Solonen 2013). Contaminants pass into feathers only during their formation, making the level of contamination in feathers an optimal bioindicator of the overall contamination near nesting sites.

### Lead in the feathers of *Sternidae* and *Laridae*

Estimates regarding lead concentrations in the feathers of water birds in East Asia demonstrate significant contamination in such countries as Korea, Iran and China (Table 1). In black-tailed gull (*Larus crassirostris*) chicks from Hongdo Island, Gyeongsangnam-do (Korea), feathers showed a lead concentration of 3.24 ± 1.75 μg/g dry weight (dw) (Kim and Oh 2014a). In the population from the islands of Seomando and Dokdo, the concentration of lead in feathers was 2.47 μg/g dw and 2.52 μg/g dw (Kim et al. 2013), respectively. In chicks and adult black-tailed gulls from Chilsando Island (Jeollanam-do, Korea), the level of lead in feathers was 0.74 ± 0.35 μg/g dw and 2.02 ± 0.69 μg/g dw (Kim and Oh 2015b), respectively. In the population of Saunders’s gulls (*Larus saundersi*) in Dongtai, Jiangsu province (China), the concentration of lead in feathers was 2.05 ± 0.47 μg/g dw (Fu et al. 2014). In the area of Rishiri Island (Japan), the concentration of lead in feathers of black-tailed gulls was measured at 0.374 ± 0.220 μg/g dw in a 10-year-old study (Agusa et al. 2005). Among the siberian gull (*Larus heuglini*) population in the Hara Biosphere Reserve of Southern Iran, a country with a very active oil industry, the concentration of lead in feathers was 7.04 μg/g dw (Mansouri et al. 2012b).

**Table 1:** The concentration of lead in the feathers of the *Laridae* and *Sternidae*

Species	Concentration (μg/g dw)	SD	Country	References
Asia				
Saunders’s gull (*Larus saundersi*)	2.05	0.47	China (Dongtai, Jiangsu province)	Fu et al. 2014
Black-tailed gull (*Larus crassirostris*)	2.02	0.69	Korea (Chilsando Island, Jeollanam-do)	Kim and Oh 2015b
Black-tailed gull (*Larus crassirostris*)	0.374	0.220	Japan (Rishiri Island)	Agusa et al. 2005
Siberian gull (*Larus heuglini*)	7.04		Iran (Hara Biosphere Reserve)	Mansouri et al. 2012b
USA				
Grey-backed tern (*Onychoprion lunatus*)	0.942	0.312	USA (Midway)	Burger and Gochfeld 2000
Franklin’s gull (*Leucophaeus pipixcan*)	2.86	0.67	USA (Agassiz National Wildlife Refuge, MN)	Burger and Gochfeld 1996b
Herring gull (*Larus argentatus*)	4.10	0.26	USA (Long Island, NY)	Burger 1995
Glaucous-winged gulls (*Larus glaucescens*)	0.855	0.133	USA (AK)	Burger et al. 2009
Europe				
Audouin’s gull (*Ichthyaetus audouinii*)	1.365	0.518	Spain (The Llobregat Delta)	García-Tarrasón et al. 2013
Yellow-legged gull (*Larus michahellis*)	0.2		Spain (The National Park of the Galician Atlantic Islands)	Moreno et al. 2011
Ivory gull (*Pagophila eburnea*)	0.13	0.01	Norway (Svalbard)	Lucia et al. 2016

Lead concentrations in the feathers of water birds in the USA varied significantly depending on the studied area and species. In the feathers of the brown noddy (*Anous stolidus*) collected in Midway, the measured concentration of lead was 0.289 ± 0.063 μg/g dw (Burger and Gochfeld 2000). In feathers from the sooty tern (*Sterna fuscata*), the Pb level was 0.519 ± 0.048 μg/g dw. In the same study, the concentrations of lead in grey-backed terns (*Onychoprion lunatus*) and white terns (*Gygis alba*) were 0.942 ± 0.312 μg/g dw and 1.380 ± 0.693 μg/g dw, respectively. The levels of lead concentration among the brown noddy (*Anous stolidus*) and grey-backed tern (*Onychoprion lunatus*) populations nesting in Midway were 0.289 ± 0.063 μg/g dw and 0.942 ± 0.312 μg/g dw, respectively (Burger et al. 2001). In studies conducted in the 1990s, the concentration of lead in feathers of herring gulls (*Larus argentatus*) from Long Island, NY (USA) was 4.10 ± 0.26 μg/g dw (Burger 1995). In Franklin’s gulls (*Leucophaeus pipixcan*) in Agassiz National Wildlife Refuge, MN (USA), the concentration of lead was 2.86 ± 0.67 μg/g dw (Burger and Gochfeld 1996b). In studies of glaucous-winged gulls (*Larus glaucescens*) from the clean areas of the Aleutian Islands in Alaska (USA), the concentration of this heavy metal was 0.855 ± 0.133 μg/g dw (Burger et al. 2009). In Prince William Sound, AK, among the population of black-legged kittiwakes (*Rissa tridactyla*), the concentration of lead was 0.707 ± 0.131 μg/g dw (Burger et al. 2008).

Compared to China or Korea, European countries are generally much less polluted with lead. Despite the 2002 “Prestige” tanker oil spill, the concentration of lead in the feathers of yellow-legged gulls (*Larus michahellis*) in the National Park of the Galician Atlantic Islands on the north-western coast of Spain measured 1 year later was 1.2 μg/g dw and in 2007 as low as 0.2 μg/g dw (Moreno et al. 2011). On the south-east coast of Spain in the Ebro Delta and the Llobregat Delta, in Audouin’s gull (*Ichthyaetus audouinii*) chick feathers, the concentrations of lead were 0.298 ± 0.065 μg/g dw and 1.365 ± 0.518 μg/g dw, respectively (García-Tarrasón et al. 2013). In Svalbard (Norway) in the Arctic Ocean, the concentration of lead in ivory gull (*Pagophila eburnea*) feathers ranged from 0.08 ± 0.01 μg/g dw in 2011 to 0.13 ± 0.01 μg/g dw in 2012 (Lucia et al. 2016), indicating an area with negligible lead contamination from anthropogenic sources.

The aforementioned numbers demonstrate that certain Asian countries (i.e. China, Korea and Iran) are the most highly polluted with lead, overall. The average feather lead levels in *Sternidae* and *Laridae* living in these countries are greater than 2 μg/g dw due to highly concentrated industrialization and poor environmental protection. Much more desirable lead levels can be found in Japan, probably due to its favorable location on the Pacific Ocean. On the other hand, European countries (Spain) and the USA show lower levels of lead pollution. However, more research on European and American populations of *Sternidae* and *Laridae* is required to accurately assess the state of the environment there.

### Lead in the feathers of *Anatidae*

Studies of the feathers of the *Anatidae* indicate high levels of contamination in Korea (Table 2). In the case of the spot-billed duck (*Anas poecilorhyncha*) and white-fronted goose (*Anser albifrons*) from Gimpo, Gyeonggi-do (Korea), measured lead concentrations in feathers were 1.69 ± 1.54 μg/g dw and 1.96 ± 1.04 μg/g dw, respectively (Kim and Oh 2014c). On the other side of the Yellow Sea, the areas around Swan Lake, Rongcheng, China are more polluted. The feathers of wintering whooper swans (*Cygnus cygnus*) contained 3.64 ± 1.13 μg/g dw of lead (Wang et al. 2017). Pakistan has much lower lead pollution levels than China, but the level is still higher than satisfactory. In common pochard (*Aythya ferina*) feathers from Baroghil valley (sparsely populated areas) and Soan valley (densely populated areas), the concentration of lead was 0.91 ± 0.03 μg/g dw and 1.97 ± 0.57 μg/g dw, respectively (Abbasi et al. 2015). In contrast, the common teal (*Anas crecca*) showed higher lead levels of 1.19 ± 0.74 μg/g dw (Baroghil valley) and 2.34 ± 0.31 μg/g dw (Soan valley) (Abbasi et al. 2015). In north-western Iran, the lead level in mallard feathers was 0.71 ± 0.15 μg/g dw (Mansouri and Majnoni 2014). Iran’s areas are diverse in terms of lead contamination. The southern part of the Caspian Sea in northern Iran is heavily polluted, with feather lead concentrations in mallards (*Anas platyrhynchos*), pintail ducks (*Anas acuta*) and greylag geese (*Anser anser*) of 2.02 ± 3.13 μg/g dw, 3.05 ± 3.51 μg/g dw and 0.81 ± 1.16 μg/g dw, respectively (Karimi et al. 2016).

**Table 2:** The concentration of lead in the feathers of the *Anatidae*

Species	Concentration (μg/g dw)	SD	Country	References
Asia				
White-fronted goose (*Anser albifrons*)	1.96	1.04	Korea	Kim and Oh 2014c
Whooper swans (*Cygnus cygnus*)	3.64	1.13	China (Swan Lake, Rongcheng)	Wang et al. 2017
Common pochard (*Aythya ferina*)	1.97	0.57	Pakistan (Soan valley)	Abbasi et al. 2015
Common pochard (*Aythya ferina*)	0.91	0.03	Pakistan (Baroghil valley)	Abbasi et al. 2015
Mallard (*Anas platyrhynchos*)	2.02	3.13	Iran (Caspain Sea)	Karimi et al. 2016
Mallard (*Anas platyrhynchos*)	0.71	0.15	Iran (north-western)	Mansouri and Majnoni 2014
USA				
Common eider (*Somateria mollissima*)	0.530	0.066	USA (AK)	Burger et al. 2008
Canada goose (*Branta canadensis*)	0.488	0.075	USA (Agassiz National Wildlife Refuge, MN)	Burger and Gochfeld 1996a
Europe				
Mute swan (*Cygnus olor*)	1.11	1.23	Hungary (Keszthely Bay, Lake Balaton)	Grúz et al. 2015
Mallard (*Anas platyrhynchos*)	0.45		Poland (Zator)	Binkowski and Sawicka-Kapusta 2015

In comparison, the USA is only lightly contaminated. In Alaska, in the Aleutians (USA), the concentration of lead in the feathers of the common eider (*Somateria mollissima*) was 0.530 ± 0.066 μg/g dw (Burger et al. 2008). In Agassiz National Wildlife Refuge, MN (USA), lead concentration in the feathers of Canada geese (*Branta canadensis*) was also low, at 0.488 ± 0.075 μg/g dw (Burger and Gochfeld 1996a).

In Europe, in mute swans (*Cygnus olor*) caught at Keszthely Bay, Lake Balaton, Hungary, the level of lead in feathers was 1.11 ± 1.23 μg/g dw (Grúz et al. 2015). In Poland near Zator, a Natura 2000 area and at the Milicz ponds nature reserve in Milicz, the concentrations of lead in mallard (*Anas platyrhynchos*) feathers were 0.45 μg/g dw and 0.18 μg/g dw, respectively (Binkowski and Sawicka-Kapusta 2015).

The quoted values confirm high levels of lead pollution in China, Korea, Pakistan and Iran. Lead levels in Anatidae feathers were often above 1.5 μg/g dw. European countries and the USA are less contaminated as shown by lead levels in Anatidae feathers below 1 μg/g dw. However, more studies showing current lead contamination in central North America and in many European countries are required.

### Lead in the feathers of *Ardeidae*

The *Ardeidae* are a family of water birds residing in wetlands and inland water areas and feeding on fish and invertebrates (Mansouri et al. 2012a). They are commonly found in warmer regions of the Northern Hemisphere. Due to their position at the top of the food chain, they are particularly vulnerable to heavy metal intoxication. Because of the similarities in nutrition, members of this family are good comparative bioindicators of wetlands and waters of the warmer regions of the Northern Hemisphere.

In China, in the areas surrounding industrialised cities in the province of Jiangsu, the concentrations of lead in the feathers of the little egret (*Egretta garzetta*) and great egret (*Egretta alba*) in a recent study were 4.55 ± 0.96 μg/g dw and 5.15 ± 4.62 μg/g dw, respectively (Table 3) (Fu et al. 2014). Among the population of black-crowned night herons (*Nycticorax nycticorax*), the lead concentration was 5.28 ± 2.22 μg/g dw (Fu et al. 2014). At the beginning of the 1990s, the degree of contamination in Hong Kong was at a similar level, as supported by studies showing a lead concentration in the feathers of great egrets (*Egretta alba*) of 4.80 ± 0.67 μg/g dw (Burger and Gochfeld 1993). In nestlings of this species, the contamination level was 1.5 ± 0.4 μg/g dw, indicating an increasing accumulation of lead with age (Burger and Gochfeld 1993). Among some *Ardeidae* nestlings in Hong Kong, the concentration of lead in feathers was higher. In the population of cattle egrets (*Bubulcus ibis*) and little egrets (*Egretta garzetta*), the levels were 4.6 ± 0.4 μg/g dw and 4.4 ± 0.6 μg/g dw, respectively (Burger and Gochfeld 1993). Lead contamination among black-crowned night heron (*Nycticorax nycticorax*) nestlings was 9.1 ± 2.2 μg/g dw (Burger and Gochfeld 1993). More recent studies show a decrease in lead contamination levels near Hong Kong. In feathers collected in 2000 from little egrets (*Egretta garzetta*), lead concentrations ranged from 0.8 to 4.4 μg/g dw, depending on where the feathers were collected (Connell et al. 2002). In other regions of China, as mentioned previously, environmental lead pollution remains high. At the beginning of the 1990s, near the city of Quiqchenqshan in the Sichuan province in China, the concentrations of lead in the feathers of nestlings of the pond heron (*Ardeola grayii*) and the black-crowned night heron (*Nycticorax nycticorax*) were 4.2 ± 1.0 μg/g dw and 5.6 ± 0.7 μg/g dw, respectively (Burger and Gochfeld 1993).

**Table 3:** The concentration of lead in the feathers of the *Ardeidae*

Species	Concentration (μg/g dw)	SD	Country	References
Asia				
Little egret (*Egretta garzetta*)	4.55	0.96	China (Jiangsu)	Fu et al. 2014
Little egret (*Egretta garzetta*)	2.65	0.76	Korea (Gyeonggi-do)	Kim and Oh 2014d
Cattle egret (*Bubulcus ibis*)	43.1	13.4	Pakistan (Mailsi)	Ullah et al. 2014
Western reef heron (*Egretta gularis*)	4.22		Iran (Hara biosphere reserve)	Mansouri et al. 2012b
North America				
Great egret (*Ardea alba*)	0.0543	0.0173	USA (Barnegat Bay; NY)	Burger 2013
Cattle egret (*Bubulcus ibis*) (nestling)	0.247		Cuba	Rodríguez et al. 2013
Europe				
Little egret (*Egretta garzetta*) (chick)	0.087	0.097	Spain (Izero island)	Rubio et al. 2016

Studies performed using bird feathers from Korea and Pakistan also indicate high levels of environmental contamination with lead. In little egret (*Egretta garzetta*) and grey heron (*Ardea cinerea*) nestlings from the city of Pyeongtaek in Gyeonggi-do (Korea), the concentrations of lead in feathers were 2.65 ± 0.76 μg/g dw and 2.05 ± 1.27 μg/g dw, respectively (Kim and Oh 2014d). In the same region, the level of lead contamination among nestlings of the black-crowned night heron (*Nycticorax nycticorax*) was 2.57 ± 1.49 μg/g dw (Kim and Oh 2014d). Pakistan appears to be significantly more contaminated with lead. Studies of lead concentration in the feathers of the cattle egret (*Bubulcus ibis*) in the areas of Lahore and Sialkot indicate a contamination level of 297 ± 11 μg/g dw and 286 ± 18 μg/g dw, respectively (Abdullah et al. 2015). Lead contamination in the areas surrounding the towns of Shorkot and Mailsi is also high. The concentrations of this heavy metal in the feathers of the cattle egret (*Bubulcus ibis*) were 32.5 ± 10.3 μg/g dw (Shorkot) and 43.1 ± 13.4 μg/g dw (Mailsi) (Ullah et al. 2014). Lead concentrations in the feathers of the cattle egret (*Bubulcus ibis*) near the Chenab River and Ravi River were as high as 37.5 ± 10.7 μg/g dw and 76.5 ± 8.6 μg/g dw, respectively (Malik and Zeb 2009). In the population of cattle egrets (*Bubulcus ibis*) near Rawal Lake reservoir site, not far from Islamabad City, the level of lead was 60.2 ± 20.7 μg/g dw (Malik and Zeb 2009).

The areas of Iran are currently at a similar level of lead contamination. Studies around the Hara biosphere reserve showed that the concentration of lead in the feathers of the western reef heron (*Egretta gularis*) was 4.22 μg/g dw (Mansouri et al. 2012b).

In comparison to the aforementioned countries, the level of lead contamination around the USA is relatively low. In the 1990s, in Agassiz National Wildlife Refuge in the state of Minnesota, the concentrations of lead in the feathers of the American bittern (*Botaurus lentiginosus*) and the black-crowned night heron (*Nycticorax nycticorax*) were 1.110 ± 0.309 μg/g dw and 0.671 ± 0.105 μg/g dw (Burger and Gochfeld 1996a), respectively. In Barnegat Bay, NY (USA), the lead concentration in the feathers of great egrets (*Ardea alba*) in 1989 was 1.460 ± 0.765 μg/g dw and has systematically become lower since that time (Burger 2013). In 1996, the concentration of lead in the feathers of this species was 0.886 ± 0.234 μg/g dw, and in 2011, the level was 0.0543 ± 0.0173 μg/g dw (Burger 2013).

In Cuba, in the nestlings of the cattle egret (*Bubulcus ibis*) and tricolored heron (*Egretta tricolor*), the concentrations of lead in feathers were 0.247 μg/g dw and 0.296 μg/g dw, respectively (Rodríguez et al. 2013).

A similar lead content in Ardeidae feathers was found in European countries. In little egret chicks on Izero Island (north coast of Spain) and Enmedio Island (south coast of Spain), the levels of lead were 0.087 ± 0.097 μg/g dw and 0.462 ± 0.824 μg/g dw, respectively (Rubio et al. 2016). In a study conducted in the late 1990s in Italy, close to the town of Pavia, among the little egret (*Egretta garzetta*) and black-crowned night heron (*Nycticorax nycticorax*) populations, the concentrations of lead in feathers were 4.52 μg/g dw and 3.36 μg/g dw, respectively (Fasola et al. 1998).

Studies on *Ardeidae* feathers can be helpful in studying the distribution of lead contamination in the Northern Hemisphere. They demonstrate that various areas of Asia (China, Korea, Pakistan, Iran) have since the 1990s until now shown high levels of pollution (lead concentration above 2 μg/g dw). Opposite results are shown by studies conducted in North American and European countries. In the populations of *Ardeidae* in these countries, the concentration of lead in feathers has consistently been below 1 μg/g dw. However, further environmental studies are still needed to show the current and accurate state of the environment in these countries.

### Lead in the bones of water birds

Due to their high level of calcium in the form of phosphates, bones accumulate xenobiotics, including heavy metals. However, the level of heavy metals, including lead, in bones does not indicate a temporary state of the animal, but the average exposure throughout its whole lifetime. Therefore, toxicological studies of bone materials from animals allow for the analysis of the average state of the natural environment over several years prior to the collection of the studied material (Hać and Krechniak 1996; Conti et al. 2012; Nascimento et al. 2016; Winiarska-Mieczan and Kwiecień 2016). This makes it possible to exclude the effect of periodic fluctuations in the studied xenobiotic levels caused by the season, the weather or the chance of the animal hunting down a source of food particularly contaminated with lead.

In Gimpo, Gyeonggi-do (Korea), the concentrations of lead in the bones of mallards (*Anas platyrhynchos*) and spot-billed ducks (*Anas poecilorhyncha*) were measured at 10.6 ± 11.1 μg/g dw and 10.30 ± 6.94 μg/g dw, respectively (Kim and Oh 2014e). In the same area, in the population of white-fronted geese (*Anser albifrons*), lead concentration was 0.93 ± 1.22 μg/g dw (Kim and Oh 2014e). In Korea, the *Ardeidae* also shown high concentrations of lead in bones. In the bones of nestlings of the grey heron (*Ardea cinerea*) and the black-crowned night heron (*Nycticorax nycticorax*) from Pyeongtaek, the levels of this heavy metal were 2.60 ± 1.11 μg/g dw and 4.71 ± 3.29 μg/g dw, respectively (Kim and Oh 2015a). In the case of the nestlings of the intermediate egret (*Egretta intermedia*) and the little egret (*Egretta garzetta*), the lead concentrations were 1.17 ± 0.90 μg/g dw and 1.26 ± 1.36 μg/g dw, respectively (Kim and Oh 2015a).

In Maryland (USA), in the 1960s, at the time when leaded petrol and paint were used, the concentrations of lead in the populations of surf scoters (*Melanitta perspicillata*) and wood ducks/carolina ducks (*Aix sponsa*) were 5.06 μg/g dw and 5.86 μg/g dw, respectively (Bagley and Locke 1967). In the case of the mallard (*Anas platyrhynchos*) and the Canada goose (*Branta canadensis*), the concentrations were 13.3 μg/g dw and 2.66 μg/g dw, respectively (Bagley and Locke 1967). In Galveston Bay, TX (USA), at the end of the 1970s, the concentrations of lead in the populations of cattle egrets (*Bubulcus ibis*) and laughing gulls (*Larus atricilla*) were 10.57 ± 5.12 μg/g dw and 9.24 ± 1.23 μg/g dw (Hulse et al. 1980). Other studies of the fauna of this area show that among royal terns (*Thalasseus maximus*) and sandwich terns (*Thalasseus sandvicensis*), the concentrations of lead in bones were 3.28 ± 1.69 μg/g dw and 1.49 ± 3.31 μg/g dw, respectively (Maedgen et al. 1982).

In Europe, the concentration of lead in the bones of waterbirds varies greatly. In south-eastern Spain, in the areas of El Hondo, in marbled teals (*Marmaronetta angustirostris*) and white-headed ducks (*Oxyura leucocephala*), the concentrations of lead in bones were 5.19 μg/g dw and 91.75 μg/g dw (Taggart et al. 2009). The territory of Poland, also shows a considerable variety in pollution levels. In bones of adult mallards (*Anas platyrhynchos*) in the north-western part of Poland around the city of Szczecin and the Słońsk Waterfowl Reserve, the concentration of lead was 5.908 ± 6.70 μg/g dw and 1.574 ± 1.863 μg/g dw, respectively (Kalisińska et al. 2004). Near Zator, a Natura 2000 area, and Milicz pond nature reserve in Milicz, the concentration of lead in bones of mallards was 1.94 μg/g dw and 0.41 μg/g dw, respectively (Binkowski and Sawicka-Kapusta 2015).

Data concerning the concentrations of lead and other heavy metals in the bones of birds are very rare due to the difficulty of catching these animals. Moreover, birds are legally protected in many areas, making the data on lead concentrations in birds’ bones very partial and incomplete. However, data on bone levels in birds is particularly valuable because it corresponds to long-term exposure and as such, it is worth conducting more such studies in the future.

### Lead in the liver of water birds

Once it enters into the organism, lead is collected and accumulated by all tissues (Winiarska-Mieczan 2014; Takano et al. 2015; Nascimento et al. 2016; Winiarska-Mieczan and Kwiecień 2016). However, it is very quickly removed from soft tissues, such as the liver or kidneys. For this reason, studies of soft tissues only reflect the current state of lead contamination and the current degree of exposure. Results of studies of soft tissues should be analyzed with great caution because a temporary increase in lead contamination caused by atmospheric factors or a small ecological catastrophe can significantly influence the results, and these results may be unrepeatable. A year apart, in the same study, the results often show completely different levels of lead contamination.

### Lead in the liver of the *Laridae* and *Sternidae*

Studies of lead levels in the livers of the *Laridae* and *Sternidae* confirm that the state of the environment has significantly improved over the last 40 years (Table 4). In the 1970s, in the population of laughing gulls (*Leucophaeus atricilla*) from West Galveston Bay, TX (USA), the concentration of lead in livers was 18.0 ± 13.0 μg/g dw among males and 12.0 ± 8.0 μg/g dw among females (Munoz et al. 1976). In a different study of adult laughing gulls (*Leucophaeus atricilla*) in the same area, the level of lead in the liver was 17.70 ± 2.47 μg/g dw (Hulse et al. 1980). In the case of prefledglings and downy young, the concentrations of lead in the liver were 10.4 ± 0.8 μg/g dw and 6.13 ± 1.93 μg/g dw, respectively (Hulse et al. 1980). Similar results were found in an analysis of tern livers. In adult royal terns (*Thalasseus maximus*), the concentration of lead in the liver was 1.433 ± 0.333 μg/g dw (Maedgen et al. 1982). Among prefledglings and downy young, the concentrations were 0.600 ± 0.133 μg/g dw and 0.633 ± 0.133 μg/g dw, respectively (Maedgen et al. 1982). In adult sandwich terns (*Thalasseus sandvicensis*), the level of lead in the liver was 1.767 ± 0.667 μg/g dw (Maedgen et al. 1982). The values for prefledgling and downy young sandwich terns (*Thalasseus sandvicensis*) were 0.800 ± 0.166 μg/g dw and 0.833 ± 0.167 μg/g dw (Maedgen et al. 1982). The northern areas of the USA are presently relatively uncontaminated. Among the black guillemot (*Cepphus grylle*) and the thick-billed murre (*Uria lomvia*) in Baffin Bay (Canada), the concentrations of lead in the liver were 0.110 ± 0.043 μg/g dw and 0.303 ± 0.440 μg/g dw, respectively (Borgå et al. 2006). In Northern Baffin Bay (Canada), the concentration of lead in the liver of kittiwakes (*Rissa* sp.) was 0.057 ± 0.030 μg/g dw (Borgå et al. 2006). Also, studies of samples of glaucous gull (*Larus hyperboreus*) livers from 1983 and 1992 show that lead levels in Baffin Bay (Canada) have been below 0.09 μg/g dw for a while (Braune and Scheuhammer 2008).

**Table 4:** The concentration of lead in the liver of the *Laridae* and *Sternidae* across years

Species	Concentration (μg/g dw)	SD	Country	References
Asia				
Black-tailed gull (*Larus crassirostris*)	2.02	0.69	Korea (Chilsando Island, Jellanam-do)	Kim and Oh 2017
Black-tailed gull (*Larus crassirostris*)	0.022	0.009	Japan (Rishiri Island)	Agusa et al. 2005
Siberian gull (*Larus heuglini*)	5.1	0.8	Iran (Hara forests of Qeshm)	Hoshyari et al. 2012
North America				
Laughing gull (*Leucophaeus atricilla*)	17.70	2.47	USA (Galveston Bay, TX)	Hulse et al. 1980
Royal tern (*Thalasseus maximus*)	1.433	0.333	USA (Galveston Bay, TX)	Maedgen et al. 1982
Kittiwake (*Rissa* sp.)	0.057	0.030	Canada (Northern Baffin Bay)	Borgå et al. 2006

Due to modern limitations on the use of lead in petrol and paint, the levels of this heavy metal in the liver of the *Laridae* are generally significantly lower in more recent measurements. In Rishiri Island (Japan), in the population of black-tailed gulls (*Larus crassirostris*), the level of lead in the liver was 0.022 ± 0.009 μg/g dw (Agusa et al. 2005). In contrast, environmental contamination with lead is still very high in some countries. In recent studies carried out in the Hara forests of Qeshm (Iran), the concentration of lead in the liver of the Siberian gull (*Larus heuglini*) was 5.1 ± 0.8 μg/g dw (Hoshyari et al. 2012). In the case of nestlings of the black-tailed gull (*Larus crassirostris*) from Hongdo Island and Rando Island (Korea), the concentrations of lead in the liver were 4.82 ± 1.80 μg/g dw and 3.71 ± 2.17 μg/g dw (Kim and Oh 2014b), respectively. In the black-tailed gull population of Chilsando Island, Jellanam-do, Korea, the concentrations of lead in adult and nestling livers were 2.02 ± 0.69 μg/g dw and 0.74 ± 0.35 μg/g dw, respectively (Kim and Oh 2017).

Due to species protection, literature data on lead concentration in *Laridae* and *Sternidae* livers are only partial. Nevertheless, available data show that in populations of these birds from areas uncontaminated with lead, concentrations of this heavy metal are below 0.5 μg/g dw. In more contaminated areas, such as South Korea or Iran, concentrations are more than ten times higher.

### Lead in the liver of the *Ardeidae*

Studies of the livers of birds from other families also indicate a high level of lead in Korea. Analysis of lead contamination in the tissues of the *Ardeidae* indicates a high degree of pollution of this country. In the case of grey herons (*Ardea cinerea*) and little egrets (*Egretta garzetta*) from the provinces of Gyeonggi-do, Chungcheongnam-do and Seoul city in South Korea, the concentrations of lead in the livers were 5.32 ± 2.01 μg/g dw and 4.19 ± 1.57 μg/g dw, respectively (Kim and Oh 2013). Among Schrenck’s bitterns (*Ixobrychus eurhythmus*), the concentration was 7.97 ± 4.38 μg/g dw (Kim and Oh 2013). Other studies show that the environment near the city of Pyeongtaek in the province of Gyeonggi-do (Korea) is also highly contaminated with lead. In black-crowned night herons (*Nycticorax nycticorax*) and grey herons (*Ardea cinerea*), the concentrations of lead in livers were 4.43 ± 2.42 μg/g dw and 3.56 ± 1.93 μg/g dw, respectively (Kim and Oh 2015a). In the population of intermediate egrets (*Egretta intermedia*) and little egrets (*Egretta garzetta*), the levels of lead were 2.98 ± 1.19 μg/g dw and 3.36 ± 1.29 μg/g dw, respectively (Kim and Oh 2015a).

India is another country which is highly contaminated with lead. In the case of the cattle egret (*Bubulcus ibis*) and the little egret (*Egretta garzetta*) from Nilgiris district, Tamil Nadu (India), the concentrations of lead in the livers were 13.26 ± 1.23 μg/g dw and 3.23 ± 1.60 μg/g dw (Jayakumar and Muralidharan 2011). These results point to a persistant and significantly higher level of lead in these areas of Asia, even in comparison to measurements taken in North America during the time when leaded petrol was used. In the 1970s, in Galveston Bay, TX (USA), lead concentration in adult specimens of the cattle egret (*Bubulcus ibis*) was 2.10 ± 0.43 μg/g dw (Hulse et al. 1980). In prefledglings and downy young, lead levels were 1.96 ± 0.40 μg/g dw and 1.03 ± 0.17 μg/g dw (Hulse et al. 1980), respectively, all noticeably lower than recent measurements in Korea and India.

Literature data show that the current level of lead in *Ardeidae* livers from South Korea is above 3 μg/g dw. This is much higher than levels in the 1970s and 1980s in the USA during the period of intensive use of leaded petrol. At that time, the level of lead in *Ardeidae* livers was around 2 μg/g dw. This clearly demonstrates the current undesirable state of the environment on the Korean peninsula.

### Lead in the liver of the *Anatidae*

The use of the *Anatidae* in bioindicative studies shows significant differences in lead contamination between various regions of Asia (Table 5). According to a 10-year-old study, the level of lead contamination in the area of the Izumi coast of Japan is relatively low, as evidenced by a lead concentration in the liver of mallards (*Anas platyrhynchos*) of 0.728 ± 0.368 μg/g dw (Nam et al. 2005). According to recent studies around the city of Gimpo (Korea), the level of lead contamination is very high there. In the population of mallards (*Anas platyrhynchos*) and spot-billed ducks (*Anas poecilorhyncha*), the concentrations of lead in the liver were 4.74 ± 2.92 μg/g dw and 4.61 ± 2.51 μg/g dw, respectively (Kim and Oh 2014e). Earlier studies by the same author show that this result is enduring, with measurements from 2 years prior showing mallards with a lead concentration of 4.24 ± 2.09 μg/g dw (Kim and Oh 2012). In the case of the white-fronted goose (*Anser albifrons*), lead contamination was 1.32 ± 2.61 μg/g dw (Kim and Oh 2014e) and 7.26 ± 6.03 μg/g dw, depending on the location in South Korea (Kim and Oh 2012). Other *Anatidae* in Korea are also characterized by a high concentration of lead in their livers. In whooper swans (*Cygnus cygnus*), the median lead level was 2 μg/g dw (Nam and Lee 2011). In mallards from north-western Iran, the concentration of lead in the liver was 1.81 ± 0.20 μg/g dw (Mansouri and Majnoni 2014). In gadwall (*Anas strepera*) and common teals (*Anas crecca*) wintering on Miankaleh and Gomishan International Wetlands, located in the southern part of Caspian Sea (northern Iran), the concentrations of lead in the liver were 10.73 and 3.60 μg/g dw (Sinkakarimi et al. 2018), respectively. Other studies confirm that the Caspian Sea is a highly polluted area—in the livers of mallards and common pochards (*Aythya ferina*), the concentrations of lead were 3.87 ± 1.37 μg/g dw and 7.87 ± 3.33 μg/g dw (Sinka-Karimi et al. 2015), respectively. At the beginning of the 1990s, in Turkey, environmental contamination with lead was also relatively high. Studies carried out in the Göksu Delta (Turkey) on the livers of mallards (*Anas platyrhynchos*) showed that the concentration of lead was 1.641 μg/g dw (Ayas and Kolankaya 1996).

**Table 5:** The concentration of lead in the liver of the *Anatidae*

Species	Concentration (μg/g dw)	SD	Country	References
Asia				
Mallard (*Anas platyrhynchos*)	0.728	0.368	Japan (Izumi coast)	Nam et al. 2005
Mallard (*Anas platyrhynchos*)	4.74	2.92	Korea (Gimpo)	Kim and Oh 2014e
Mallard (*Anas platyrhynchos*)	1.81	0.20	Iran (north-western)	Mansouri and Majnoni 2014
Mallard (*Anas platyrhynchos*)	3.87	1.37	Iran (Caspian Sea)	Sinkakarimi et al. 2018
Mallard (*Anas platyrhynchos*)	1.641		Turkey (Göksu Delta)	Ayas and Kolankaya 1996
USA				
Mallard (*Anas platyrhynchos*)	0.77	0.17	USA (Illinois River, IL)	Levengood 2003
Blue-winged teal (*Spatula discors*)	0.40	0.67	USA (Gulf of Mexico, TX)	Fedynich et al. 2007
Common eiders (*Somateria mollissima*)	0.088		USA (Table Bay, AK)	Mallory et al. 2017
Europe				
Common teal (*Anas crecca*)	2.2		France	Mateo and Guitart 2003
Marbled teal (*Marmaronetta angustirostris*)	1.5		Spain	Taggart et al. 2009
Mallard (*Anas platyrhynchos*)	0.963	2.670	Austria (eastern)	Plessl et al. 2017
Mallard (*Anas platyrhynchos*)	0.763	1.850	Poland (Szczecin)	Kalisińska et al. 2004

Studies in European countries indicate that lead contamination is still at a high level locally in certain areas. In Southwestern France, in Le Verdon-sur-Mer in Gironde and in the coastal wetlands at Hourtin, among greylag geese (*Anser anser*) the concentration of lead in livers was as high as 151.4 ± 95.8 μg/g dw (Lucia et al. 2010). Spain also shows evidence of lead contamination. Studies carried out between 1986 and 1995 on the livers of *Anatidae* collected from various places around the country showed a level of lead above 1 μg/g dw (Mateo and Guitart 2003). The aforementioned studies focused on birds such as the common pochard (*Aythya ferina*) (5.8 μg/g dw), the common teal (*Anas crecca*) (2.2 μg/g dw), the eurasian wigeon (*Anas penelope*) (1.7 μg/g dw), the gadwall (*Anas strepera*) (1.6 μg/g dw), the mallard (*Anas platyrhynchos*) (17 μg/g dw), the northern pintail (*Anas acuta*) (18 μg/g dw), the northern shoveler (*Anas clypeata*) (3.3 μg/g dw), the red-crested pochard (*Netta rufina*) (1.6 μg/g dw) and the tufted duck (*Aythya fuligula*) (6.1 μg/g dw) (Mateo and Guitart 2003). In the areas of El Hondo in south-eastern Spain, in the livers of marbled teals (*Marmaronetta angustirostris*) and white-headed ducks (*Oxyura leucocephal*), lead concentrations were 1.5 μg/g dw and 54 μg/g dw, respectively (Taggart et al. 2009). The areas of central Europe are much less polluted. In mallard livers from eastern Austria, the concentration of lead was 0.963 ± 2.670 μg/g dw (Plessl et al. 2017). In the north-western part of Poland around Szczecin and the Słońsk Waterfowl Reserve, the concentrations of lead in livers of adult mallards were 0.763 ± 1.850 and 1.14 ± 2.26 μg/g dw, respectively (Kalisińska et al. 2004). Similar concentrations were found in the population of mallards near the Zator Natura 2000 area and the Milicz ponds nature reserve in Milicz. In these cases, the concentration of lead in mallard livers was 0.48 μg/g dw (Zator) and below the detection threshold (Milicz) (Binkowski and Sawicka-Kapusta 2015). Compared to Poland or Austria, the areas of the Evros Delta in Greece, close to the border with Turkey, are more polluted with lead. However, lead levels in the livers of members of this group of waterfowl depend greatly on the species. In mallards and gadwall (*Anas strepera*), the levels of lead were 2.39 μg/g dw and 2.43 μg/g dw, respectively, while in northern shovelers (*Anas clypeata*) and common teals (*Anas crecca*), levels were 0.986 μg/g dw and 0.465 μg/g dw, respectively (Aloupi et al. 2017).

In comparison, the areas of the USA are not highly contaminated. Near Illinois River, in the state of Illinois, the concentration of lead in the liver of the mallard (*Anas platyrhynchos*) was 0.77 ± 0.17 μg/g dw (Levengood 2003). In the liver of the blue-winged teal (*Spatula discors*) from the area of the Gulf of Mexico, TX (USA), the concentration of lead was 0.40 ± 0.67 μg/g dw (Fedynich et al. 2007). In the New Jersey Meadowlands, the concentration of lead in the liver of Canada geese (*Branta canadensis*) was 0.830 ± 0.150 μg/g dw (Tsipoura et al. 2011). This situation in the USA is a result of the early implementation of the ban on the use of lead in petrol and other products of industry. In the 1960s, prior to the ban, the concentration of lead in the liver of the *Anatidae* was much higher. A study from the state of Maryland presents the following lead concentrations in the liver: the American scoter (*Melanitta americana*) 1.7 μg/g dw, the brent goose (*Branta bernicla*) 4.3 μg/g dw, the canada goose (*Branta canadensis*) 1.7 μg/g dw, the mallard (*Anas platyrhynchos*) 3.0 μg/g dw, the snow goose (*Chen caerulescens*) 4.0 μg/g dw, the surf scoter (*Melanitta perspicillata*) 3.0 μg/g dw, the velvet scoter (*Melanitta fusca*) 2.7 μg/g dw and the wood duck/carolina duck (*Aix sponsa*) 5.0 μg/g dw (Bagley and Locke 1967).

In the mute swan livers (*Cygnus olor*) collected more recently in Chesapeake Bay, USA, the level of lead was 0.41 ± 0.29 μg/g dw, despite the collection of research material from military areas (Beyer and Day 2004). Various populations in Alaska also show low levels of lead contamination. In male king eiders (*Somateria spectabilis*) and spectacled eiders (*Somateria fischeri*) caught near Barrow in the Beaufort Sea in northern Alaska, the concentrations of lead in the liver were 0.13 ± 0.09 μg/g dw and 0.19 ± 0.27 μg/g dw, respectively (Miller et al. 2016). In common eiders (*Somateria mollissima*) from Table Bay (Newfoundland & Labrador, Canada) and from Tern Island in Foxe Basin in the Arctic Circle, the level of lead in the livers of these birds was 0.088 μg/g dw and 0.181 μg/g dw, respectively (Mallory et al. 2017).

Due to the fact that Anatidae are hunted for food and sport, it is much easier to obtain research material than for *Ardeidae*, *Laridae* and *Sternidae*. Therefore, there is more available data on lead concentration in *Anatidae* livers from different parts of the world. The above data, along with the data on lead concentration in feathers of various bird families, show that the areas of Korea and Iran are significantly contaminated with lead. In birds from these countries, the concentration of lead in the liver was consistently above 1.5 μg/g dw. In contrast, in the Japanese population, it is below 1 μg/g dw, confirming low contamination levels in Japan. In the USA, the concentration of lead in the livers of the studied birds is comparable to Japan or even lower in sparsely populated areas like Alaska. Europe is diverse in terms of lead pollution, although lead levels are generally lower than in Korea or Iran, as shown by the quoted data.

### Lead in the eggs of water birds

Among birds, the place and time of the breeding period are strictly determined. It is very common for members of a given species to build its nests every year in the same place and at approximately the same time. Therefore, toxicological studies of a region can be performed on whole eggs or empty shells, as well as the comparison of acquired results with data from previous years. This helps in the evaluation of the state of the natural environment near a breeding location and in the analysis of the exposure to lead and other xenobiotics during the early stages of bird development (Jeng et al. 1997).

Among the *Anatidae* from New Jersey (USA), in the eggs of Canada geese (*Branta canadensis*), the concentration of lead measured in recent studies was 0.483 ± 0.108 μg/g dw and in eggs of mallards (*Anas platyrhynchos*), it was 0.186 ± 0.036 μg/g dw (Tsipoura et al. 2011). Eggs of Canada geese (*Branta canadensis*) from South Brother Island (New York Harbor) and Mill Rock (New York Harbor) contained 0.313 ± 0.072 μg/g dw and 0.837 ± 0.587 μg/g dw, respectively (Burger and Elbin 2015b). In the eggs of the eider (*Somateria* sp.), in the Yukon-Kuskokwim Delta, Alaska (USA), the concentration of lead was significantly higher at 3.21 ± 0.15 μg/g dw (Grand et al. 2002). However, in the same state, in the Aleutians, lead concentration in the eggs of the common eider (*Somateria mollissima*) was only 0.306 ± 0.099 μg/g dw (Burger et al. 2008).

### Lead concentrations in the eggs of *Ardeidae*

Recent studies performed on the eggs of cattle egrets (*Bubulcus ibis*) from the areas around the industrialized cities of Pakistan—Lahore and Sialkot—showed a very high concentration of lead, 47 ± 13 μg/g dw and 41 ± 8 μg/g dw, respectively (Table 6) (Abdullah et al. 2015). Around the city of Shorkot, the concentration of lead in cattle egret (*Bubulcus ibis*) eggshells was lower, at 5.40 ± 3.01 μg/g dw (Hashmi et al. 2013). These areas seem to be particularly contaminated, especially in comparison to the wetlands of the Islam Headworks and the Trimmu Headworks, where the concentrations of lead in little egret (*Egretta garzetta*) egg contents were 0.89 ± 0.25 μg/g dw and 0.84 ± 0.54 μg/g dw (Shahbaz et al. 2013). Lead concentrations in the eggs of cattle egrets (*Bubulcus ibis*) who spend their breeding period in the same uncontaminated areas were 0.37 ± 0.28 μg/g dw and 0.44 ± 0.33 μg/g dw (Shahbaz et al. 2013). In the eggshells of the cattle egret (*Bubulcus ibis*), the degree of contamination was 0.13 ± 0.30 μg/g dw for the Islam Headworks and 0.58 ± 0.88 μg/g dw in the Trimmu Headworks (Hashmi et al. 2013). In little egret (*Egretta garzetta*) eggshells, lead concentration in the Trimmu Headworks was 1.9 ± 1.3 μg/g dw and in the Islam Headworks was 1.09 ± 0.83 μg/g dw (Hashmi et al. 2013). According to a recent study of the agricultural regions surrounding the city of Mailsi and along the Jhang-Faisalabad Road in Pakistan, the concentrations of lead in eggshells of the cattle egret (*Bubulcus ibis*) were 1.34 ± 1.25 μg/g dw and 1.44 ± 1.13 μg/g dw, respectively (Hashmi et al. 2013). In the Northwestern Persian Gulf (Iran), the level of lead contamination is lower, as demonstrated by a lower concentration of this heavy metal in eggshells and eggs of the western reef heron (*Egretta gularis*)—0.172 ± 0.090 μg/g dw and 0.191 ± 0.094 μg/g dw, respectively (Khademi et al. 2015). In the Göksu Delta (Turkey), the concentration of lead in the eggs of the little egret (*Egretta garzetta*) in a study from the early 1990s was 0.341 μg/g dw (Ayas and Kolankaya 1996). In the Büyük Menderes River, among grey herons (*Ardea cinerea*), the lead content in eggs and eggshell was 13.6 ± 3.2 μg/g dw and 3.6 ± 1.4 μg/g dw, respectively (Durmaz et al. 2017).

**Table 6:** The concentration of lead in the eggs of the *Ardeidae*

Species	Concentration (μg/g dw)	SD	Country	References
Asia				
Great egret (*Egretta alba*)	82.1	6.9	China (Wuxi)	Fu et al. 2014
Little egret (*Egretta garzetta*)	0.06		China (Pearl River Delta)	Zhang et al. 2006
Little egret (*Egretta garzetta*)	0.007	0.007	China (Hong Kong)	Lam et al. 2005
Cattle egret (*Bubulcus ibis*)	47	13	Pakistan (Lahore)	Abdullah et al. 2015
Western reef heron (*Egretta gularis*)	0.191	0.094	Iran (Northwestern Persian Gulf)	Khademi et al. 2015
Grey heron (*Ardea cinerea*)	13.6	3.2	Turkey (Büyük Menderes River)	Durmaz et al. 2017
USA				
Black-crowned night heron (*Nycticorax nycticorax*)	0.039	0.011	USA (MN, Agassiz National Wildlife Refuge)	Burger and Gochfeld 1996a
Black-crowned night heron (*Nycticorax nycticorax*)	0.054	0.034	USA (New York Harbor)	Burger and Elbin 2015b

There is extensive data in recent literature concerning lead contamination in China. In the Pearl River Delta, Guangdong province, and around the lakes Poyang and Tai in the province of Jiangxi, the concentration of lead in the eggs of the little egret (*Egretta garzetta*) was very low (Zhang et al. 2006). In the Pearl River Delta, it measured 0.06 μg/g dw and in the area around the lakes Poyang and Tai, the values were 0.22 μg/g dw and 0.10 μg/g dw (Zhang et al. 2006). In the province of Jiangsu, in birds nesting around the cities of Wuxi, Xinghua, Dongtai and Sheyang, the concentration of lead in *Ardeidae* eggshells was very high, about 80 μg/g dw (Fu et al. 2014). A similar degree of environmental lead contamination was observed around the city of Wuxi, where lead concentrations in the eggshells of the little egret (*Egretta garzetta*), the great egret (*Egretta alba*) and the black-crowned night heron (*Nycticorax nycticorax*) were 88.5 ± 14.6 μg/g dw, 82.1 ± 6.9 μg/g dw and 84.9 ± 6.9 μg/g dw, respectively (Fu et al. 2014). Conversely, in Hong Kong, the concentrations of lead in the eggs of the black-crowned night heron (*Nycticorax nycticorax*) and the little egret (*Egretta garzetta*) were very low, at the level of 0.007 ± 0.007 μg/g dw and 0.014 ± 0.015 μg/g dw (Lam et al. 2005).

As shown previously, areas of the USA are not heavily polluted with lead, resulting in low levels of lead in eggs. In Agassiz National Wildlife Refuge, MN (USA), the concentration of lead in eggs of the black-crowned night heron (*Nycticorax nycticorax*) was 0.039 ± 0.011 μg/g dw (Burger and Gochfeld 1996a). The concentrations of lead in black-crowned night heron (*Nycticorax nycticorax*) eggs on South Brother Island (New York Harbor) and Mill Rock (New York Harbor) were 0.370 ± 0.141 μg/g dw and 0.054 ± 0.034 μg/g dw, respectively (Burger and Elbin 2015b).

Studies of egg shells and whole eggs show a very large variation in lead contamination within individual countries. An example of this is Pakistan, where, in the most polluted areas, the concentration of lead in eggs of *Ardeidae* reaches as much as 40 μg/g dw, while in other areas of the country, it is closer to 5 μg/g dw, and in the least polluted areas, as low as 0.4 μg/g dw. A similar situation can be seen in China. In areas near to large cities, the concentration of lead in eggs is very high, while other areas show medium and low lead contamination. However, studied areas across the USA, as shown in other subsections of this paper, are relatively unpolluted with lead.

### Concentration of lead in the eggs of *Laridae and Sternidae*

A significant amount of data has also been provided by studies on eggshells of the *Laridae* and *Sternidae* (Table 7, Table 8)*.* One such study estimated the level of lead in the eggs of herring gulls (*Larus argentatus*) from Long Island, NY (USA) (Burger and Gochfeld 1995). In 1989, the concentration of lead in the eggshells of these birds was 2.54 μg/g dw. In subsequent years, the concentration of this heavy metal decreased to under 1 μg/g dw, 0.773 μg/g dw in 1991 and only 0.38 μg/g dw in 1994 (Burger and Gochfeld 1995). Studies performed at the beginning of the twenty-first century in New Jersey (USA) showed that the concentration of lead in the eggs of the common tern (*Sterna hirundo*) ranged from 0.022 to 0.528 μg/g dw, depending on the place where the eggs were collected and the breeding season. Lead concentrations in common tern eggs collected from Mike’s Island in New Jersey (USA) in the years 2000 and 2002 were 0.100 ± 0.021 μg/g dw and 0.022 ± 0.004 μg/g dw, respectively (Burger and Gochfeld 2003). In the area of Barnegat Bay, NJ (USA), the concentrations of lead in the eggs of the herring gull (*Larus argentatus*) and the great black-backed gull (*Larus marinus*) were 0.273 ± 0.069 μg/g dw and 0.227 ± 0.075 μg/g dw (Burger 2002). In the case of the common tern (*Sterna hirundo*) and the Forster’s tern (*Sterna forsteri*), the concentrations were 0.164 ± 0.025 μg/g dw and 0.056 ± 0.007 μg/g dw, respectively (Burger 2002). In the eggs of the Franklin’s gull (*Leucophaeus pipixcan*) in Agassiz National Wildlife Refuge, MN (USA), the concentration of lead was 0.129 ± 0.016 μg/g dw (Burger and Gochfeld 1996b). In marsh elder in the USA, in research from 2002, the concentration of lead in the eggs of this species was 0.528 ± 0.069 μg/g dw, whereas in the year 2000, the concentration was 0.142 ± 0.020 μg/g dw (Burger and Gochfeld 2003). However, more recent studies show a significant decrease in lead pollution. An example of this is the herring gull (*Larus argentatus*) population study from Mill Rock in the New York/New Jersey Harbor Estuaries, where lead concentration in eggs in 2011 and 2012 was 0.040 ± 0.024 μg/g dw and 0.451 ± 0.095 μg/g dw, respectively (Burger and Elbin 2015a). Also, in great black-backed gulls (*Larus marinus*), the concentration of lead in eggs collected at this location was 0.138 ± 0.044 μg/g dw (Burger and Elbin 2015b).

**Table 7:** The concentration of lead in the eggshells of the *Laridae* and *Sternidae*

Species	Concentration (μg/g dw)	SD	Country	References
Asia				
Black-headed gull/Saunder’s gull (*Larus saundersi*)	79	11	China (Dongtai, Jiangsu)	Fu et al. 2014
Black-tailed gull (*Larus crassirostris*)	3.10	1.36	Korea (Hongdo Island)	Kim and Oh 2014a
Black-tailed gull (*Larus crassirostris*)	0.061	0.042	Japan (Rishiri Island)	Agusa et al. 2005
Bridled tern (*Onychoprion anaethetus*)	0.260	0.049	Iran (northwestern Persian Gulf)	Khademi et al. 2015
Mediterranean gull (*Ichthyaetus melanocephalus*)	3.70	0.80	Turkey (Büyük Menderes river)	Durmaz et al. 2017
Europe				
Black-headed gull (*Chroicocephalus ridibundus*)	0.7	0.8	Poland (northern)	Kitowski et al. 2017

**Table 8:** The concentration of lead in the eggs of the *Laridae* and *Sternidae*

Species	Concentration (μg/g dw)	SD	Country	References
Asia				
Bridled tern (*Onychoprion anaethetus*)	0.010	0.021	China (Hong Kong)	Lam et al. 2005
Black-tailed gull (*Larus crassirostris*)	0.92	0.24	Korea (Hongdo Island)	Kim and Oh 2014f
Black-tailed gull (*Larus crassirostris*)	0.92	0.24	Korea (Hongdo Island)	Kim and Oh 2014f
Black-tailed gull (*Larus crassirostris*)	0.023	0.015	Japan (Rishiri Island)	Agusa et al. 2005
Bridled tern (*Onychoprion anaethetus*)	0.286	0.064	Iran (northwestern Persian Gulf)	Khademi et al. 2015
Mediterranean gull (*Ichthyaetus melanocephalus*)	13.6	2.4	Turkey (Büyük Menderes river)	Durmaz et al. 2017
USA				
Franklin’s gull (*Leucophaeus pipixcan*)	0.129	0.016	USA (Agassiz National Wildlife Refuge, MN)	Burger and Gochfeld 2003
Herring gull (*Larus argentatus*)	0.451	0.095	USA (Mill Rock New York/New Jersey Harbor Estuaries)	Burger and Elbin 2015a
Glaucous-winged gull (*Larus glaucescens*)	0.107	0.029	USA (Aleutians, AK)	Burger et al. 2009
Europe				
Common tern (*Sterna hirundo*)	0.249		Greece (Axios Delta)	Goutner et al. 2001

Low levels of lead concentration were also observed in studies performed in the Aleutians, AK (USA). The lead content of the eggs of the glaucous-winged gull (*Larus glaucescens*) during the first decade of the twenty-first century was 0.107 ± 0.029 μg/g dw (Burger et al. 2009).

Studies of lead content in the eggs of *Laridae* show a high level of contamination in industrialized areas of Korea or in the province of Jiangsu in China. According to the most recent study, lead concentrations in the eggshells and egg contents of the black-tailed gull (*Larus crassirostris*) from Hongdo Island (Korea) were 3.10 ± 1.36 μg/g dw and 0.92 ± 0.24 μg/g dw, respectively (Kim and Oh 2014a, 2014f). In the case of the eggshells of the Chinese black-headed gull/Saunder’s gull (*Larus saundersi*) near the city of Dongtai, in the province of Jiangsu (China), the concentration of lead is as high as 79 ± 11 μg/g dw (Fu et al. 2014). In contrast, in the population of bridled terns (*Onychoprion anaethetus*) in Hong Kong, the concentration of lead in egg contents in the end the twentieth century was 0.010 ± 0.021 μg/g dw (Lam et al. 2005). In Rishiri Island (Japan), the concentrations of lead in the egg contents and eggshells of the black-tailed gull (*Larus crassirostris*) were 0.023 ± 0.015 μg/g dw and 0.061 ± 0.042 μg/g dw, respectively (Agusa et al. 2005). In the northwestern Persian Gulf in Iran, in bridled tern (*Onychoprion anaethetus*) egg contents and eggshells, lead concentrations were 0.286 ± 0.064 μg/g dw and 0.260 ± 0.049 μg/g dw (Khademi et al. 2015), respectively. This shows that Iran’s regions, despite a significant oil industry, have a lesser degree of pollution compared to China or Korea. The areas around the Büyük Menderes river in Turkey are more polluted. In eggshells and eggs of the Mediterranean gull (*Ichthyaetus melanocephalus*) found near the mouth of the river, Pb levels were 3.70 ± 0.80 μg/g dw and 30.0 ± 8.8 μg/g dw (Durmaz et al. 2017), respectively. In the vicinity of the sources of this river, lead levels were 3.6 ± 0.8 μg/g dw (eggshells) and 13.6 ± 2.4 μg/g dw (eggs) (Durmaz et al. 2017).

Studies performed in 1997 in the Evros Delta (Greece) on the eggs of the yellow-legged gull (*Larus cachinnans*) and the Mediterranean gull (*Larus melanocephalus*) revealed concentrations of 0.204 μg/g dw and 0.060 μg/g dw, respectively (Goutner et al. 2001). In the case of the common tern (*Sterna hirundo*) in Axios Delta (Greece), lead concentration was 0.249 μg/g dw (Goutner et al. 2001). In eggshells of the black-headed gull (*Chroicocephalus ridibundus*) in northern Poland, the concentration of lead ranged from 0.4 ± 0.1 to 0.7 ± 0.8 μg/g dw, depending on the egg collection site (Kitowski et al. 2017).

The concentration of lead in *Laridae* and *Sternidae* eggs collected from the USA and Europe shows a relatively low level of contamination in these areas. However, the level of lead in eggs varies considerably depending on the breeding season. Because of this, results obtained from the analysis of eggs can be very accurate about the current state of the environment. Studies of eggs collected in Asia indicate high lead contamination in countries, such as Turkey, Korea and China, and relatively low contamination in Japan.

### Lead concentration in the eggs of the *Laridae* and *Sternidae* of Barnegat Bay (USA) as a bioindicator of contamination with heavy metals

Due to the precise knowledge of the time and place of breeding, bird eggs are an important material in environmental studies. For this reason, comparative studies were performed to analyze changes in lead concentration in the eggs of *Laridae* and *Sternidae* over severals years in Barnegat Bay, NJ, USA (Table 9). These studies show a pattern of decreasing lead contamination in the studied area. In the breeding period of 1971, lead concentration in tern eggs was 8.64 ± 2.56 μg/g dw (Burger and Gochfeld 1988). In 1982, the concentration was reduced to 1.338 ± 0.234 μg/g dw (Burger and Gochfeld 1988). Twenty years later, the degree of lead contamination in the eggs of the *Laridae* and *Sternidae* in Barnegat Bay was more than two times lower (Burger 2002; Burger and Gochfeld 2003), between 0.528 ± 0.069 and 0.022 ± 0.009 μg/g dw, depending on the species and the exact breeding location. Such differences in the results can be explained by irregularity in the level of lead contamination of Barnegat Bay, which is filled by waters flowing from numerous rivers and is where local water is exchanged with ocean water (Burger and Gochfeld 2003). Moreover, seasonal fluctuations in lead concentrations in bird eggs may be related to natural factors, such as increased strength of storms. Another important factor influencing time-related differences in lead concentration may be human activity, including accidental catastrophes—which can lead to environmental contamination with lead—or periodic changes in industrial activity.

**Table 9:** The concentration of lead in the eggs of the *Sternidae* coming from the breeding regions in New Jersey Bay, USA. The data is presented according to the years of research

Species	Concentration (μg/g dw)	SD	Years	References
Tern (*Sternidae*)	8.64	2.56	1971	Burger and Gochfeld 1988
Tern (*Sternidae*)	1.338	0.243	1982	Burger and Gochfeld 1988
Common tern (*Sterna hirundo*)	0.164	0.025	2000	Burger 2002
Forster’s tern (*Sterna forsteri*)	0.056	0.007	2000	Burger 2002
Common tern (*Sterna hirundo*)	0.142	0.020	2000	Burger and Gochfeld 2003
Common tern (*Sterna hirundo*)	0.502	0.232	2001	Burger and Gochfeld 2003
Common tern (*Sterna hirundo*)	0.164	0.025	2001	Burger and Gochfeld 2003
Common tern (*Sterna hirundo*)	0.528	0.069	2002	Burger and Gochfeld 2003

The quoted data show unambiguously that the level of lead contamination has decreased over the last 50 years. In the 1970s, Pb levels in the eggs of *Sternidae* living in Barnegat Bay exceeded 8 μg/g. In the 1980s, following the withdrawal of leaded petrol and paints, the level of contamination in eggs fell to 1 μg/g dw. In the years following, the level of lead contamination decreased even further, to the point where, in 2000, it amounted to a maximum of 0.5 μg/g.

## Conclusion

The development of industry and demographic changes of the last 20 years have been accompanied by unfavorable changes in the natural environment, including air, water and soil pollution with heavy metals, such as lead. Despite the introduction of a number of restrictions on the use of lead, its concentration in the environment remains high, particularly in certain heavily industrialized and populated areas, leading to long-term human and animal exposure and lead toxicity. Monitoring the state of the environment through the use of indicator organisms can provide valuable information on the actual impact of newly introduced environmental protection measures.

Due to the ability of birds to travel long distances in the air, the potential feeding area of each individual is much larger than that of typical terrestrial animals. This makes birds a convenient indicator of environmental lead pollution over large areas, in particular areas of inland and coastal waters.

Based on data from the many wild bird species belonging to the families of *Anatidae*, *Ardeidae*, *Sternidae* and *Laridae*, we were able to make an approximate assessment of the state of the entire aquatic ecosystem of the Northern Hemisphere in terms of Pb pollution over a period of the last 20 years.

Based on research on the different organs, feathers and eggs of water birds, it can be concluded that lead is present at varying levels in the environment across the Northern Hemisphere. In the Far East, the highest levels of lead pollution can be observed in China and Korea, due to their extreme industrialization. In Japan, the level of lead in the environment is much lower due to the preference of Japanese industry for safe, advanced technology which uses less natural resources and has less destructive impact on the environment. This is further aided by the country’s favourable location on the Pacific Ocean. India and Pakistan, as well as oil-producing countries like Iran, also contain areas heavily polluted with lead. India and Pakistan are densely populated, highly industrialized and have high levels of car traffic. According to bioindication studies, areas with active oil industries (such as Iran) are also heavily polluted with this heavy metal. Western Europe (Spain, France, Italy) is less polluted with lead in comparison. Nevertheless, the level of pollution in these European countries is greater than satisfactory, despite the introduction of a number of bans restricting the use of lead, for example, the use of leaded petrol or lead-containing paints. Lead does not decompose and so it can persist in the environment for many years after its use has ceased. Finally, the USA and Canada appear to be the areas with the lowest level of lead pollution. This may be due to the low population density in these countries and a high concern for the environment, encouraging the use of modern technologies that do not require the use of this element. Abandoning the use of lead in petrol has also been crucial in reducing environmental lead contamination. As a result, the current level of lead pollution in the USA is much lower than in the 1970s and 1980s. However, due to the fragmented nature of the data, it is difficult to determine any such a trend across Europe.
